# The complete mitochondrial genome of the least horseshoe bat (*Rhinolophus pusillus*)

**DOI:** 10.1080/23802359.2020.1717389

**Published:** 2020-01-27

**Authors:** Jiaying Wang, Ao Zhao, Haijian Sun

**Affiliations:** a Institute of Estuarine and Coastal Research, East China Normal University, Shanghai, China;; b School of Ecological and Environmental Sciences, East China Normal University, Shanghai, China

**Keywords:** Bat, mitochondrial genome, *de novo* assembly, *Rhinolophus pusillus*

## Abstract

In this study, we generated the complete mitochondrial genome of *Rhinolophus pusillus* using next-generation sequencing. The mitochondrial genome was 16,833 bp in length and contained 13 protein-coding genes, 22 tRNA, 2 rRNA, and a non-coding control region. Phylogenetic analyses supported the taxonomic status of *Rhinolophus pusillus* among genus *Rhinolophus*, and the grouping with the sister taxon *R. monoceros*, which was highly restricted to Taiwan Island.

The horseshoe bats are the second most speciose in bats (at least 100 recognized species; Csorba et al. 2019), and form a single genus, *Rhinolophus*. Among the genus, the least horseshoe bat (*Rhinolophus pusillus*) is the most widely distributed member throughout the Indomalayan realm, also is one of the smallest horseshoe bat with highest echolocation peak frequency occurred in this area (Zhang et al. 2009; Csorba et al. 2019). In contrast, the sister taxon *R. monoceros* is endemic to Taiwan Island. Both species are members of pusillus group in genus *Rhinolophus* (Li et al. 2006). Here, we generated and annotated the complete mitochondrial genome of *Rhinolophus pusillus* (GenBank Accession number: MN820985). Phylogenetic analysis confirmed that *R. pusillus* and *R. monoceros* formed a monophyletic group.

An adult male of *R. pusillus* was collected from Anhui province in China (30°26′51.82″N, 118°26′54.16″E). The 3-mm wing membrane was obtained and kept in 95% ethanol at −80 °C laboratory freezer in East China Normal University (Voucher No. AH19). Genomic DNA was extracted using DNeasy Blood & Tissue Kit (Qiagen, Hilden, Germany). A DNA library with insert fragments of ∼300 bp was constructed and sequenced on an Illumina Hi-seq X Ten sequencer (150 bp paired-end). Raw reads were trimmed with Trimmomatic (Bolger et al. 2014) with default parameters. We performed *de novo* assembly based on all trimmed reads using SPAdes 3.1.1 (Bankevich et al. 2012). A nearly complete mitochondrial genome was generated and then automatically annotated using MitoZ (Meng et al. 2019), and also manually checked in comparison with published data (*R. rex*, NC_028536).

The complete mitochondrial genome of *R. pusillus* was 16,833 bp in length, including 13 protein-coding genes (PCGs), one 12s rRNA gene, one 16s rRNA gene, 22 transfer RNA genes (tRNA) and a non-coding control region (D-loop), and the overall base composition were 31.6% A, 28.4% C, 25.6% T, and 14.4% G. Among genes annotated, *nd6* and eight tRNA genes were encoded on the light strand, while other genes (12 PCGs, 14 tRNA genes, and 2 rRNA genes) were all located on heavy strand. The 13 protein-coding genes was 11,408 bp in length. Most PCGs were initiated with ATG except for *nd2*, *nd3*, *nd5* with ATA and *nd4l* with GTG. *Cox1*, *cox2*, *atp8*, *atp6*, nd3, *nd4l*, *nd5*, and *nd6* were terminated with TAA or TAG, while *cytb* was terminated with AGA. Besides, incomplete stop codon (T–– or TA–) was observed in *nd1*, *nd2*, *cox3*, and *nd4*.

We constructed the maximum-likelihood (ML) consensus tree based on the 13 concatenated PCGs of 14 *Rhinolophus* species and one outgroup *Hipposideros armiger* (Figure 1). The ML tree was constructed using RAxML v.8.2.11 (Stamatakis 2014) with GTRGAMMA model. Node support was estimated from 1000 replicate bootstrap searches. The ML tree clearly supported that *R. pusillus* and *R. monoceros* were sister taxon and formed a monophyletic group with 100% bootstrap support.

**Figure 1.**
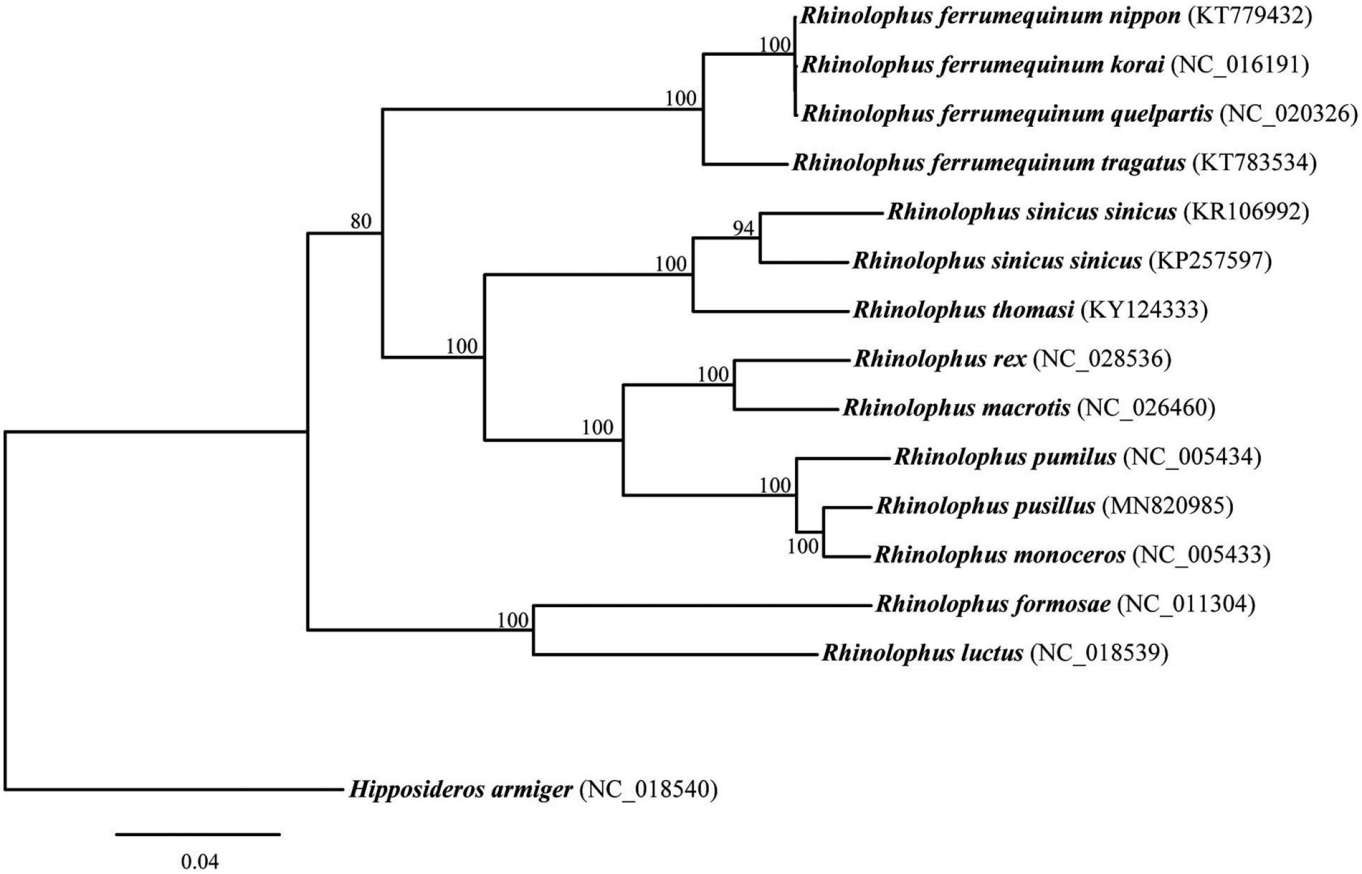
Maximum-likelihood phylogeny of 14 *Rhinolophus* bat mitogenomes. Numbers at the nodes indicated bootstrap support values.
